# Patterns of item score and total score distributions on depression rating scales in the general population: evidence and mechanisms

**DOI:** 10.1016/j.heliyon.2020.e05862

**Published:** 2020-12-28

**Authors:** Shinichiro Tomitaka

**Affiliations:** a Department of Health Promotion and Human Behavior, Kyoto University Graduate School of Medicine/School of Public Health, Kyoto, Japan; b Department of Mental Health, Panasonic Health Center, Tokyo, Japan

**Keywords:** Depression rating scales, Likert scales, Mathematical distribution, Exponential distribution, Level of measurement, Latent trait, Item response

## Abstract

Recent research has demonstrated that item scores and total scores on depression rating scales exhibit common distribution shapes in a general population. Specifically, responses to depressive symptom items show a proportional relationship between response options, except for the lower end option, whereas total scores exhibit an exponential distribution, except for the lower end of the distribution. The common mathematical distributions of item scores and total scores may help explain the scoring mechanism of a depression rating scale. This paper, therefore, discusses how the distribution shapes are generated. Two conditions are assumed: (1) each individual's latent degree of depression forms an exponential distribution in a general population, and (2) the threshold of each depressive symptom forms a normal distribution. A simulation study applying the two assumptions revealed that simulated total scores follow an exponential distribution through a strong linear relationship between an individual's latent trait of depression and simulated total scores. Furthermore, the strong linear relationship between total scores and the individual's latent trait of depression supports the prevailing view that total scores on a Likert type scale tend toward interval data. Regarding item scores, an analysis of boundary curves, which divide the distribution of total scores by each item score, revealed that the lower end option and the next option distances have a trade-off relationship, and the remaining option distances have a proportional relationship across all items. In conclusion, the assumption that a latent trait of depression follows an exponential distribution helps explain the mathematical pattern of item response and total score distribution. Furthermore, the item score and total score distribution shapes on depression rating scales may serve as evidence of the level of measurement.

## 1 Introduction

A Likert-type scale is one of the most used psychometric scales for measuring psychological conditions [1]. As a screening tool for depression, a variety of Likert type scales, such as the Center for Epidemiologic Studies Depression Scale (CES-D), the six-item Kessler Psychological Distress Scale (K6), and the nine-item Patient Health Questionnaire (PHQ-9) have been developed to measure the degree of depressive symptoms [2, 3, 4]. For inferential statistics, researchers often assume normality for such Likert type scale data. However, in the case of depression rating scales, a normal distribution is rarely observed in a general population [5, 6]. As the majority of individuals in a general population have a few or no depressive symptoms, the item score and total score distributions on such a scale are usually skewed to the right [2, 7].

Although little attention has been paid to the mathematical property of such skewed distributions [8], recent research has revealed that item score and total score distributions from depressive symptom scales exhibit a common mathematical pattern in a general population [9, 10, 11]. Specifically, total scores on these scales follow an exponential distribution, except for the lower end of the distribution [12]. Furthermore, responses to all depressive symptom items show a proportional relationship between response options, except for the lower end option [10]. These findings have been confirmed repeatedly by analyzing large-scale data collected from dozens of nationally representative surveys in the US, EU, and Japan [12, 13, 14, 15].

Generally, a probability distribution reflects how variables are generated [16]. Therefore, the shape of item score and total score distributions may lead to a better understanding of depression rating scale scoring mechanisms.

The aim of this paper is to review recent evidence regarding the mathematical patterns of item score and total score distributions from depression rating scales and discuss the mechanisms underlying these mathematical patterns. Furthermore, the levels of measurement for depression rating scales are addressed based on the mathematical patterns of item score and total score distributions.

## 2 Materials and methods

This review examined theoretical and empirical literature back to 2000 and consulted Google Scholar, PubMed, SCOPUS, PsycINFO, and EBSCO. The key search terms used to find relevant literature were “depression,” “depressive symptoms,” “exponential distribution,” “item responses,” “total scores,” “mathematical model,” and “depression rating scale.” The search identified 102 articles. The process of screening excluded 86 articles because they were not related to mathematical patterns of item score and total score distributions on depression rating scales. The remained 16 articles were considered relevant to this current review [9, 10, 11, 12, 13, 14, 15, 17, 18, 19, 20, 21, 22, 23, 24, 25]. The ethics committees of Kyoto University Graduate School of Medicine and Panasonic Health Center do not consider literature analysis to be human research and, thus, the need for ethical approval was waived.

## 3 Results

### 3.1 Patterns of total score distributions on depression rating scales

**Figure 1:**
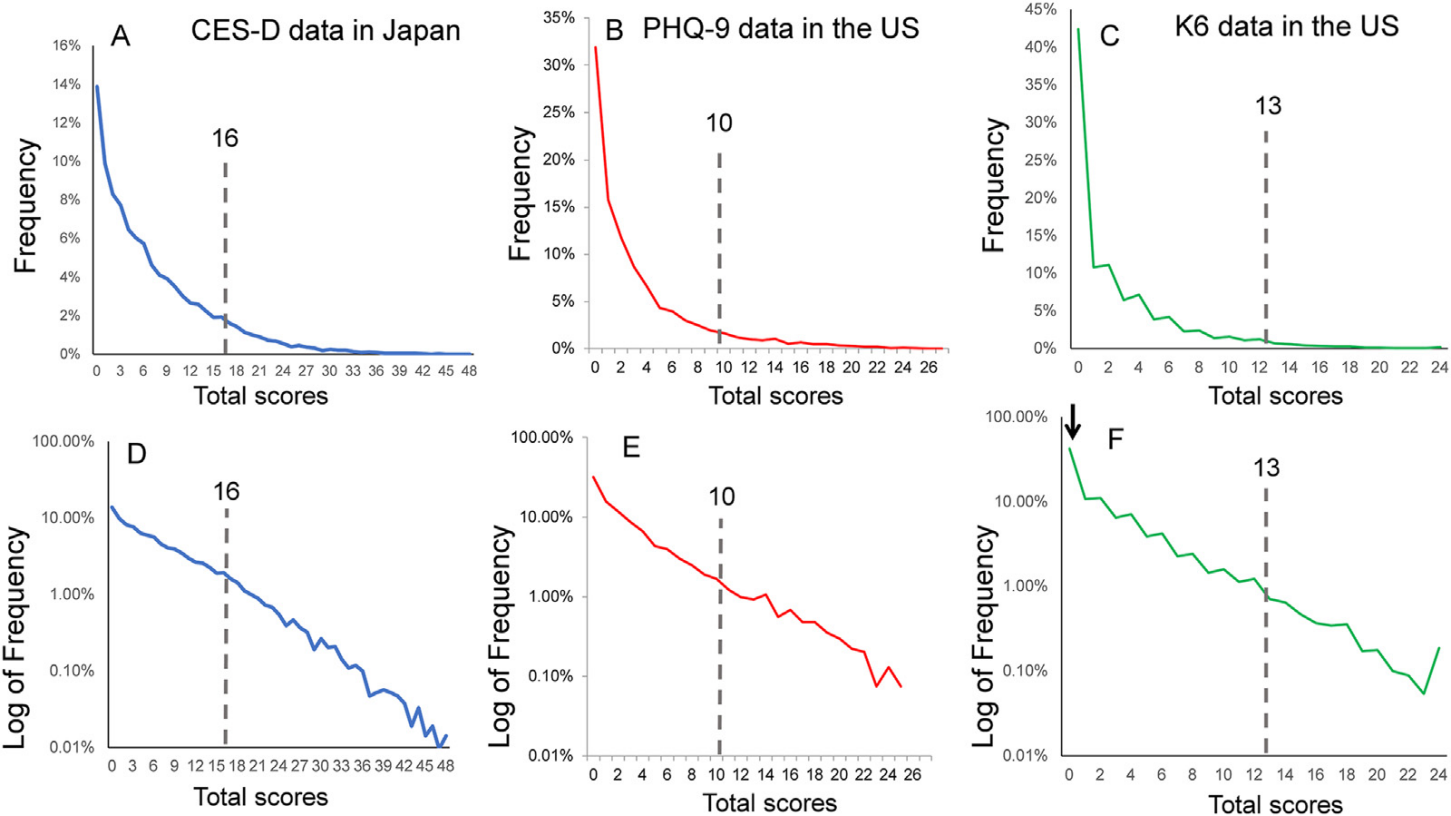
Distributions of total scores on the CES-D, PHQ-9, and K6 in the general population. (A) The distribution of the CES-D scores (sum of 16 depressive symptom item scores) from the Japanese Active Survey of Health and Welfare, (B) the distribution of PHQ-9 scores from National Health and Nutrition Examination Survey, and (C) the distribution of K6 data from the National Health Interview Survey in the US. The distributions of the CES-D, PHQ-9, and K6 total scores for the three surveys are commonly right-skewed. Auxiliary lines indicate the cut-points for clinical depression (a score of 16, 10, and 13 for the CES-D, PHQ-9 and K6, respectively). On a log-normal scale, the three distributions on the CES-D (D), PHQ-9 (E), and K6 (F) commonly show linear patterns. As indicated by the arrow, the distribution of total scores deviates from the exponential pattern at the lower end of the distribution (F). Image credit: (A) *PLoS ONE*, https://doi.org/10.1371/journal.pone.0165928.g008, (B) *BMC Psychiatry*, https://doi.org/10.1186/s12888-018-1696-9, (C) *Scientific Reports*, https://doi.org/10.1038/s41598-019-47322-1.

Generally, as a sample size increases, the shape of a sampling distribution more closely approximates a population distribution [26]. Recent analyses of large-scale data have revealed that total scores in self-rated depression scales exhibit a common distribution in the general population. For example, as shown in Figures 1A, 1B, and 1C, right-skewed distributions of total scores are similarly observed in the CES-D data from the Japanese Active Survey of Health and Welfare [12], the PHQ-9 data from the National Health and Nutrition Examination Survey [11], and the K6 data from the National Health Interview Survey in the United States (US) [14]. Generally, a score of ≧ 16 on the CES-D, a score of ≧ 10 on the PHQ-9, and a score of ≧ 13 on the K6 are defined as the cut-point for clinical depression. It appears that there is no evidence of a natural break in the total score distributions at or around the cut-point for clinical depression. On a log-normal scale, the three distributions commonly exhibit a linear pattern, except for the lower end of the distribution (Figures 1D, 1E, and 1F). As indicated by the arrow, the distribution of total scores sometimes deviates from the exponential pattern at the lower end of the distribution (Figure 1F). The exponential pattern of the three distributions covers a wide range of total scores beyond their cut-points. To our group's knowledge, although the exponential distribution of total scores has been repeatedly confirmed by dozens of nationally representative surveys in the US, Europe, and Japan, it is not observed for other types of psychological scales, such as positive affect scales [11, 12, 13].

**Figure 2:**
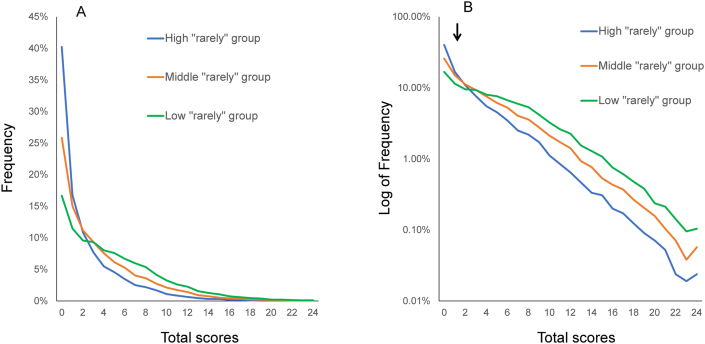
Distributions of the sum of 8 depressive symptom items for high, middle, and low “rarely” groups (A). The distributions for the three groups are right-skewed. On a log-normal scale (B), all three groups showed linear patterns in parallel over 2–8 scores. As indicated by the arrow, the distribution of the sum of 8 item scores deviates from the linear pattern at the lower end of the distribution. Image credit: *PLoS ONE*, https://doi.org/10.1371/journal.pone.0165928.g003.

Notably, previous studies demonstrated that the probability of lower-end options (e.g., “rarely” on the CES-D) predicts whether the distribution deviates either upward or downward at the lower end of the distribution [12]. Specifically, at the lower end of the distribution, the summed scores with a high probability of lower-end options deviate upward from the exponential pattern, whereas the summed scores with a low probability of lower-end options deviate downward. For example, in an analysis of CES-D data from a representative Japanese survey, the 16 depressive symptom items were grouped into three combinations according to the rank order of probability of “rarely” [12]. The high “rarely” group included items from the first to the eighth in rank order of probability of “rarely,” the middle “rarely” group included items from the fifth to the twelfth, and the low “rarely” group included items from the ninth to the sixteenth. As shown in Figure 2A, while the distributions of the summed scores for the three groups are right-skewed, the slopes at the lower end of the distributions are different from each other. On a log-normal scale, all three groups show linear patterns in parallel except for the lower end of the distribution, indicating that the three groups follow an exponential pattern with a similar rate parameter (Figure 2B). On the other hand, as indicated by the arrows in Figure 2B, the three groups exhibit individual patterns at the lower end of the distribution, indicating that patterns at the lower end of the distribution depend on the probability of item responses at the lower end.

### 3.2 Response patterns for depressive symptom items

**Figure 3:**
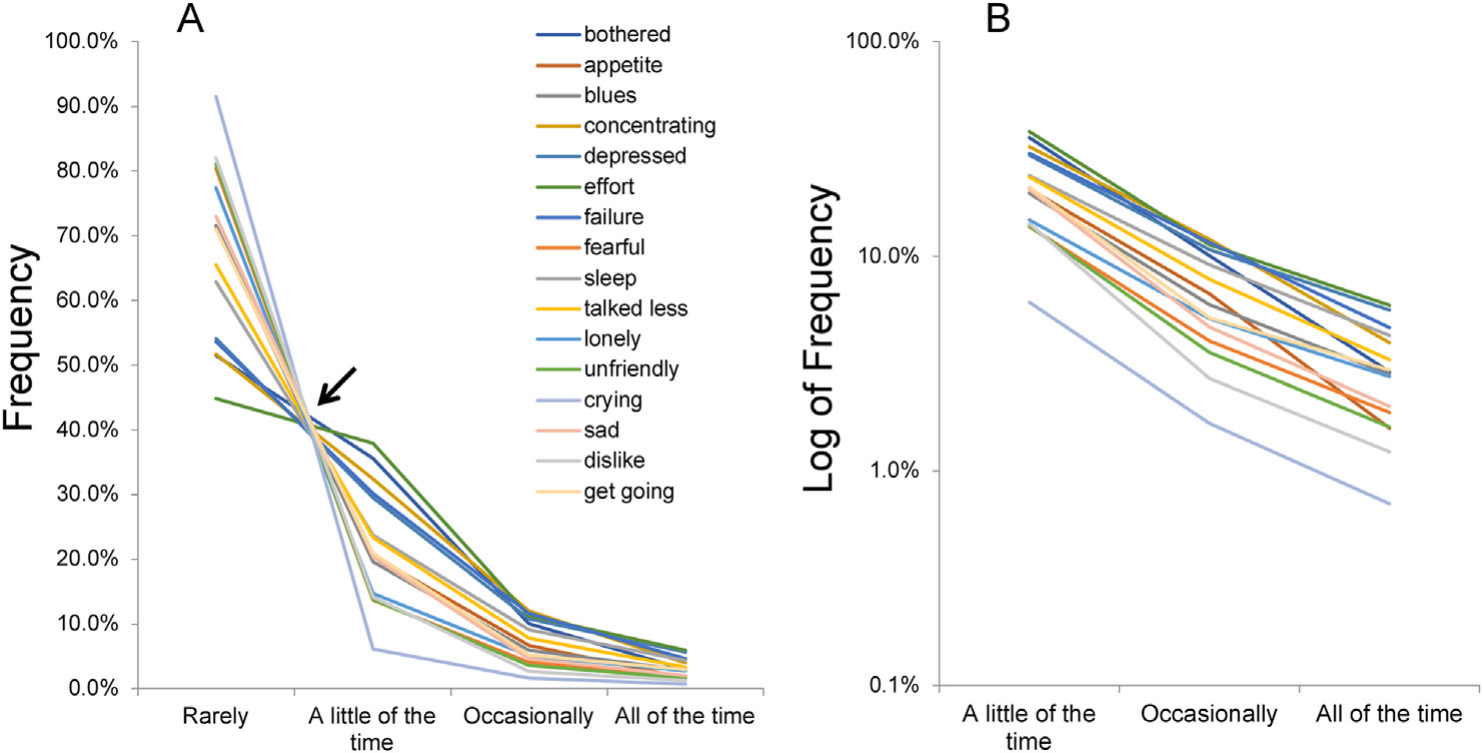
Responses to the 16 depressive symptom items on the CES-D are presented using a normal scale (A) and a log-normal scale (B). (A) Although the lines for the 16 items cross between “rarely” and “a little of the time,” they show a converging pattern for the remaining response options. (B) The lines for the 16 items decrease in parallel from “a little of the time” to “all of the time” on a log-normal scale. Image credit: *PLoS ONE*, https://doi.org/10.1371/journal.pone.0165928.g001.

Recently, analyses of large-scale survey data revealed that responses to depressive symptom items exhibit a characteristic pattern in the general population. In an analysis of CES-D data from a Japanese national survey, we found that responses to 16 depressive symptom items exhibited a common pattern [10]. As shown in Figure 3A, lines of item responses cross almost at a single point between “rarely” (score = 0) and “a little of the time” (score = 1). The lines display a converging pattern for the remaining response options. On a log-normal scale, the converging lines show a parallel pattern (Figure 3B).

Moreover, such a common characteristic pattern of item responses has been replicated for several nationally representative surveys in Europe, the US, and Japan: CES-D data from the Irish Longitudinal Study on Ageing [13], GHQ-12 data from Eurobarometer [27], PHQ-9 data from the National Health and Nutrition Examination Survey in the US [11], and K6 data from the National Health Interview Survey in the US [28].

Previous studies have shown that this characteristic pattern of item responses results from a trade-off relationship between the lower end option and the next option, and a proportional relationship between the remaining response options across all depressive symptom items [10, 28]. For example, this characteristic pattern of item responses is similar to a share of the profits between a boss and their henchmen. When they divide the profits, the boss takes a large proportion and the henchmen proportionally divide the remaining profits according to their rank. In the case of item responses, the lower-end option on a depression rating scale corresponds to the boss, and the remaining options correspond to the henchmen.

**Figure 4:**
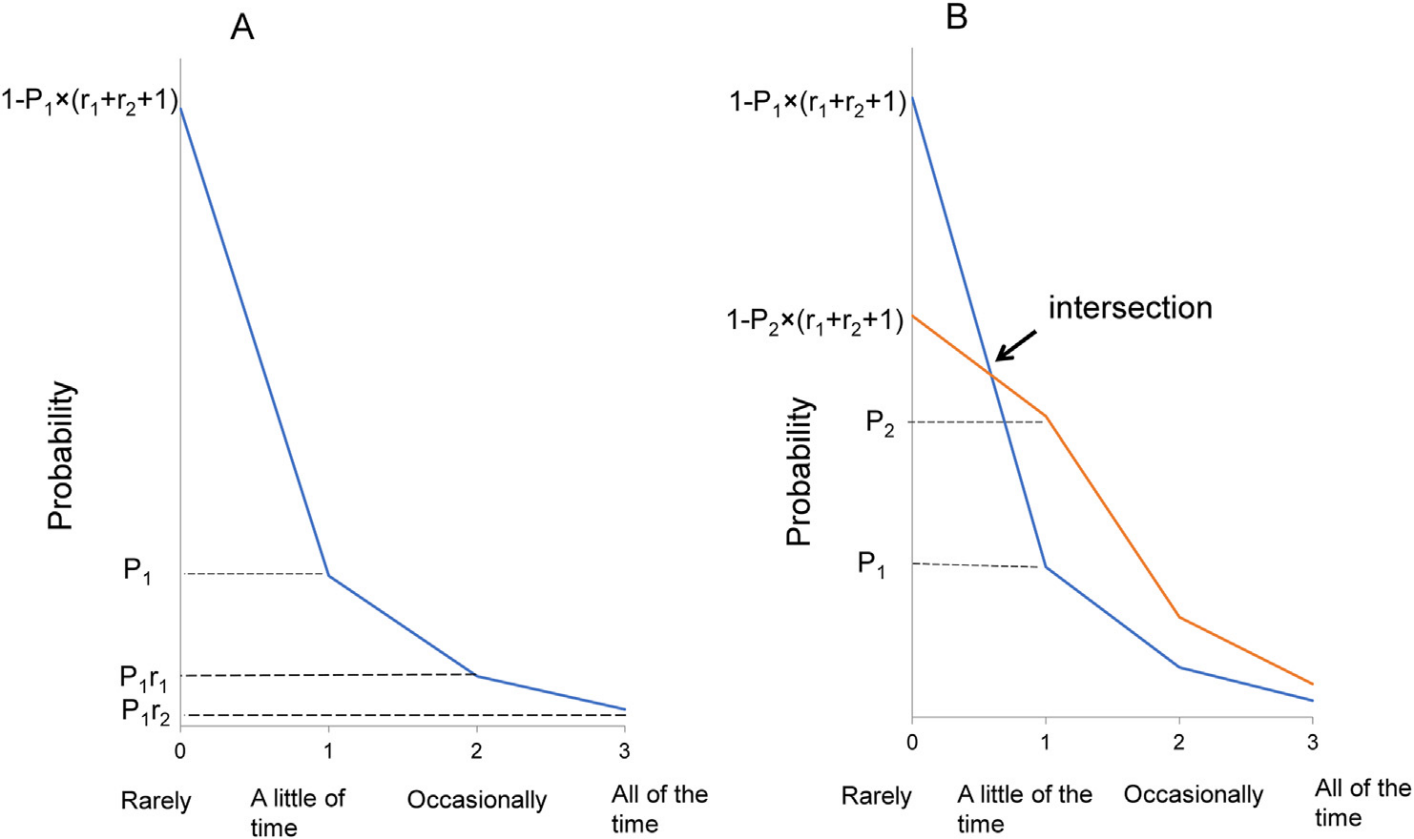
This mathematical model assumes that a proportional relationship exists between “a little of the time,” “occasionally,” and “all of the time” across all 16 items. When the probability of “score = 1” is presented as Pi (i = item number) and the ratios among “score = 1,” “score = 2,” and “score = 3” are presented as 1:r_1_:r_2_, the probabilities of “score = 1,” “score = 2,” “score = 3,” and “score = 0” are expressed as Pi, Pir_1_, Pir_2_, and 1 – Pi × (1 + r_1_ + r_2_), respectively. Image credit: *Heliyon*, https://doi.org/10.1016/j.heliyon.2019.e01387.

Based on these findings, we proposed a mathematical model of item responses on depressive symptom scales (Figure 4) [28]. On a 4-point scale (0-1-2-3) such as the CES-D, when the probability of “score = 1” is presented as P_1_ and the ratios among “score = 1,” “score = 2,” and “score = 3” are presented as 1:r_1_:r_2_, the probabilities of “score = 0,” “score = 1,” “score = 2,” and “score = 3” are expressed as 1 − P_1_ × (1 + r_1_ + r_2_), P_1_, P_1_r_1_, and P_1_r_2,_ respectively. As shown in Figure 4A, the probabilities of “score = 0,” “score = 1,” “score = 2,” and “score = 3” in another line (red line) are expressed as 1 – P_2_ × (1 + r_1_ + r_2_), P_2_, P_2_r_1_, and P_2_r_2_, respectively. Mathematically, this item response model allows all response lines to cross at a single point between the lower-end option and the next option and shows a parallel pattern for the remaining options on a log-normal scale (Figure 4B) [28].

**Figure 5:**
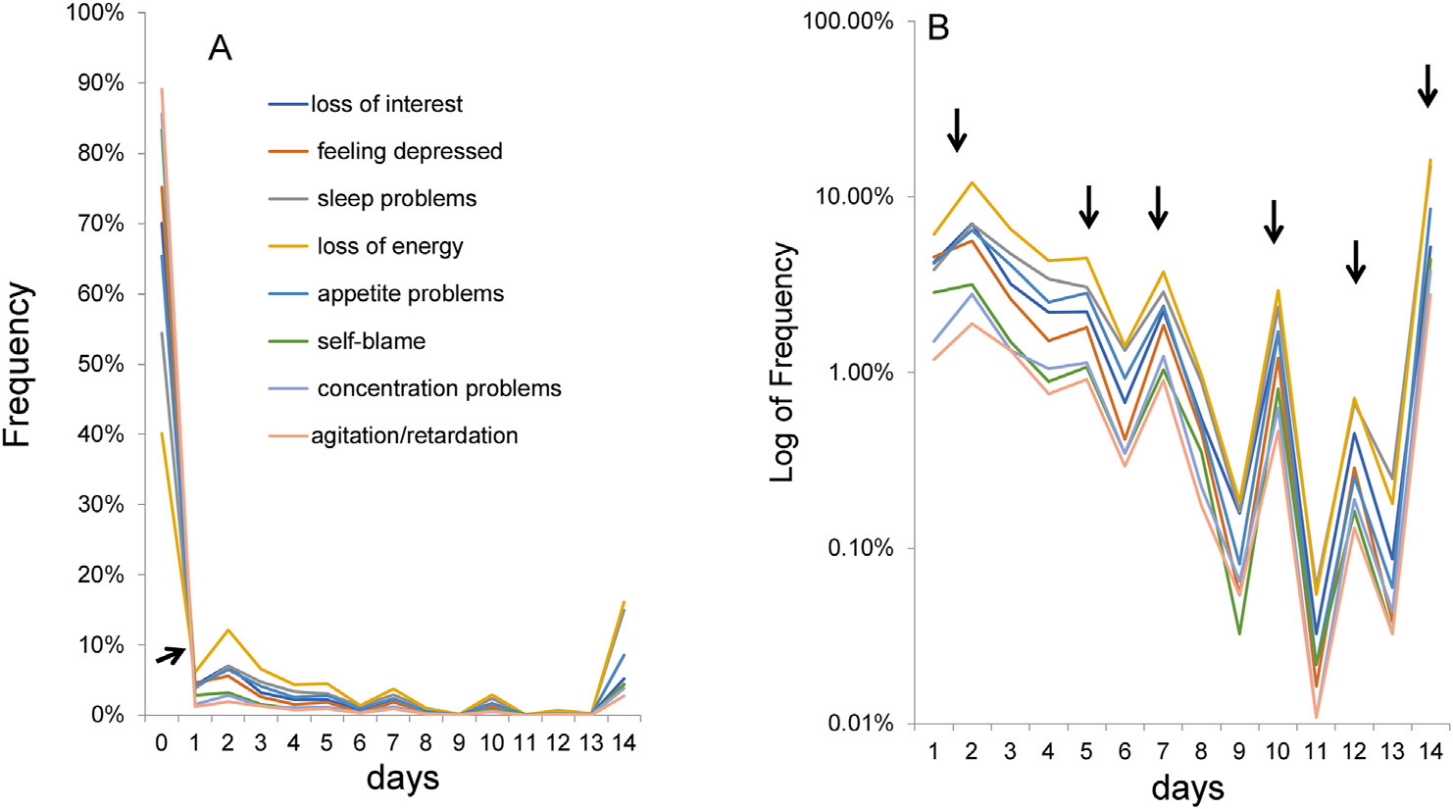
Item responses scored on a 15-point scale (0 days–14 days). (A) As indicated by the arrow, while the lines of the eight items cross at a single point between 0 days and 1 day, they fluctuate in synchrony from 1 to 14 days. (B) On a log-normal scale, the eight lines fluctuate in parallel from 1 to 14 days. As the arrows indicate, the lines show peaks at 2, 5, 7, 10, 12, and 14 days because of the influence of end-digit preference. Image credit: Frontiers in Psychiatry, https://doi:10.3389/fpsyt.2017.00251.

The model of item responses is applicable to depression rating scales with a greater number of response options. In an analysis of PHQ-8 data from the Behavioral Risk Factor Surveillance Survey in the US, respondents were asked to indicate the days of each depressive symptom in the past 14 days on a 15-point scale (0 days–14 days). As shown in Figure 5, lines of all item responses cross at a single point between “0 days” and “1 day” (Figure 5A), whereas the lines fluctuate in synchrony from 1 day to 14 days [22]. Using a log-normal scale, the lines of all item responses show a parallel fluctuation from 1 day to 14 days (Figure 5B). Although the item response pattern on the 15-point scale is complex, it is consistent with the pattern of a 4-point scale (Figure 1) in that the response lines cross at a single point between the lower end option and the next option, and the remaining options show a parallel pattern on a log-normal scale.

### 3.3 Mechanisms for an exponential pattern of total score distributions

Why do total scores on depression rating scales approximate an exponential pattern in a general population? This question can be divided into two parts, “What do total scores represent?” and “How does the distribution of total scores reflect the distribution of an individual's latent trait of depression?

First, we address the issue of what total scores on depressive symptom scales represent. In theory, Likert scaling presumes the existence of a latent continuous variable of the measured object, and Likert scale total scores are considered to represent the degree of a latent trait of the measured object [1]. Moreover, the latent trait of the measured object is generally believed to follow a normal distribution. Although Likert's theory is difficult to confirm because latent traits are not measurable, it is currently common practice to regard Likert-type total scores as the degree of a measured object.

In the case of depression rating scales, total scores are expected to represent the degree of a latent trait of depression. Furthermore, since total scores on depression scales follow an exponential pattern, the latent trait of depression is assumed to follow an exponential distribution. To my knowledge, Likert himself did not consider the possibility that a latent trait on a Likert scale might follow an exponential distribution [1, 29].

Regarding the number of Likert scale latent traits, a single latent trait is generally assumed based on the positive correlations among items [1]. Several lines of evidence suggest that the measured object of a depression rating scale is unidimensional, not multidimensional [30, 31].

Next, we address the issue of how the distribution of total scores reflects the distribution of an individual's latent trait of depression. To my knowledge, there is no established model of how each Likert-type item provides a discrete score from a continuous latent variable. Therefore, our group proposed a process model of how the individual's latent trait of depression is processed into each item score [23].

In general, measurement is the process of applying measuring instruments to measured objects [32]. We consider that Likert-type scaling is a kind of bioassay: individuals are not only the object of measurement but also a measuring tool. In bioassays, a stimulus is applied to subjects and the subjects’ response is measured [33]. In the case of a depression rating scale, a stimulus corresponds to a latent trait of depression and a response corresponds to a depressive symptom. Of note, in bioassays, the threshold of a response to the same stimulus varies among subjects. Although all individuals use the same set of response options for each depressive symptom item, each individual has a distinct threshold for each depressive symptom. For example, even with the same degree of a latent trait of depression, some individuals exhibit appetite loss and others do not. Thus, the threshold for each response option will exhibit its own distribution according to the degree of a latent trait of depression. If the threshold of each response option is assumed to exhibit a normal distribution, the response rate to each response option will follow a cumulative distribution function of normal distribution. This idea is also used in item response theory (IRT), which assumes a model of the relationship between a latent trait and the response to each response option [34]. Although IRT was originally developed using a cumulative distribution function of a normal distribution, recent IRT usually applies a logistic model because a cumulative distribution function of a normal distribution is difficult to apply [34].

**Figure 6:**
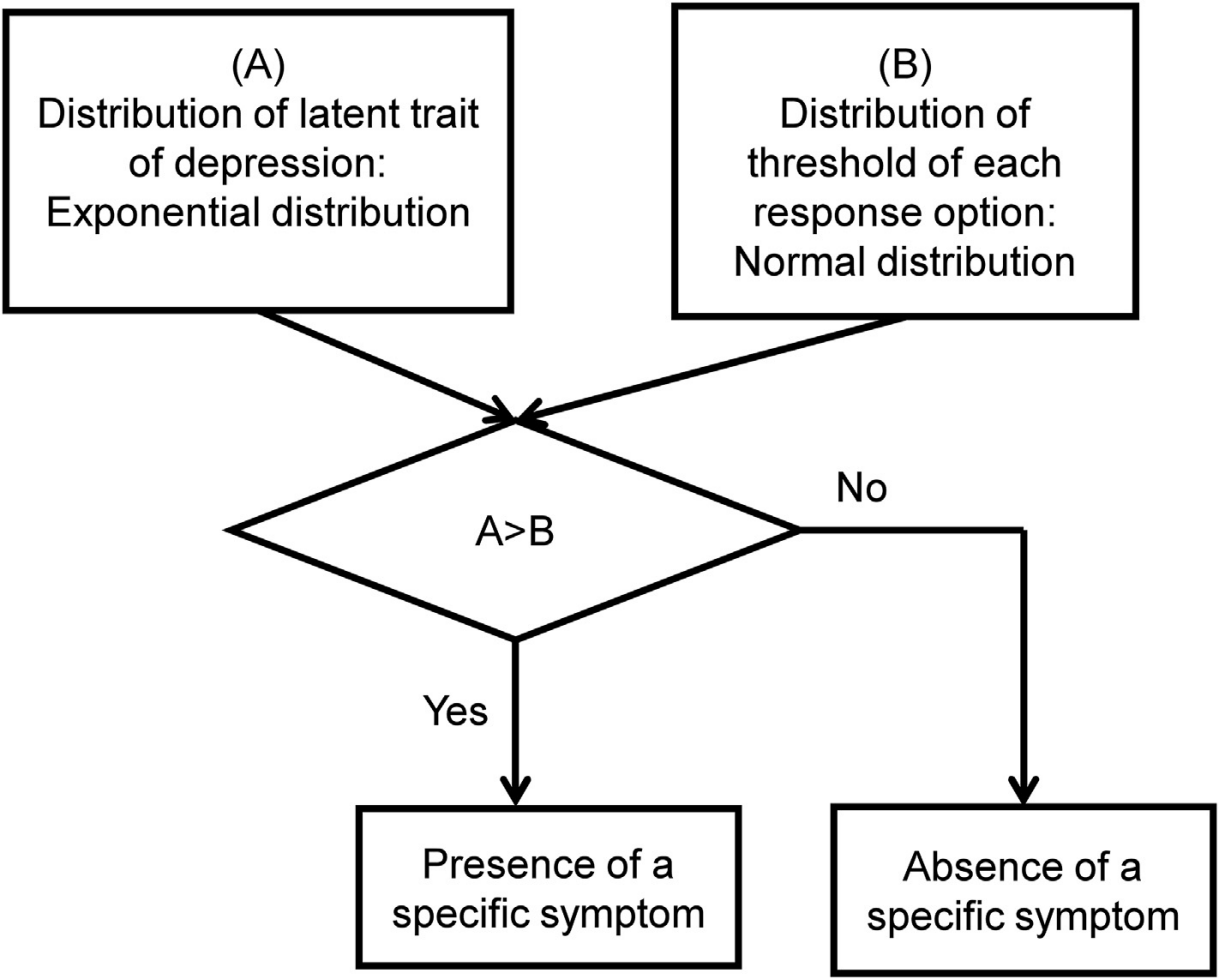
Process model of a depression rating scale. Two conditions are assumed: (1) each individual's degree of a latent trait of depression forms an exponential distribution in the general population, and (2) the threshold of self-rated depression scale scoring also forms a distribution. When each individual's severity of depression is greater than those of item thresholds for each response option, individuals choose the presence of specific response options for each depressive symptom. Image credit: *BMC Research Notes*, https://doi.org/10.1186/s13104-017-2937-6.

Figure 6 illustrates a process model of how the individual's latent trait of depression is processed into an item score. As shown in Figure 6, two conditions are assumed: (1) each individual's degree of a latent trait of depression, which varies from individual to individual, forms an exponential distribution in the general population, and (2) the threshold of self-rated depression scale scoring forms a normal distribution. When each individual's severity of depression is greater than the item threshold for each response option, individuals choose the presence of specific response options for each depressive symptom.

To confirm whether this process model can explain exponential patterns of total scores, we conducted a simulation study of depressive symptom rating [23]. As a format for the simulated scoring of depressive symptoms, the Revised Clinical Interview Schedule (CIS-R) questionnaire was used. The CIS-R, which has been used for national surveys in the UK, is a binary rating scale to measure degrees of depressive and neurotic symptoms [35]. Since the CIS-R contains 57 items, the simulated scores of CIS-R range from 0 to 57.

In line with a process model, two sets of random numbers were generated: one expressing the degree of each individual's severity of depression and another expressing each individual's threshold for each item. The mean value of the distribution of each item's threshold was set to the percentile point that was equivalent to each item's prevalence rate. Allocated mean values that corresponded to distributions of all item thresholds were not equal intervals. Random numbers of each individual's latent trait of depression greater than those of item thresholds indicated the presence of specific symptoms. We simulated 10,000 sets of random variables to approximate the number of subjects used in such a population survey. The applied simulation methods have been reported in detail elsewhere [23].

**Figure 7:**
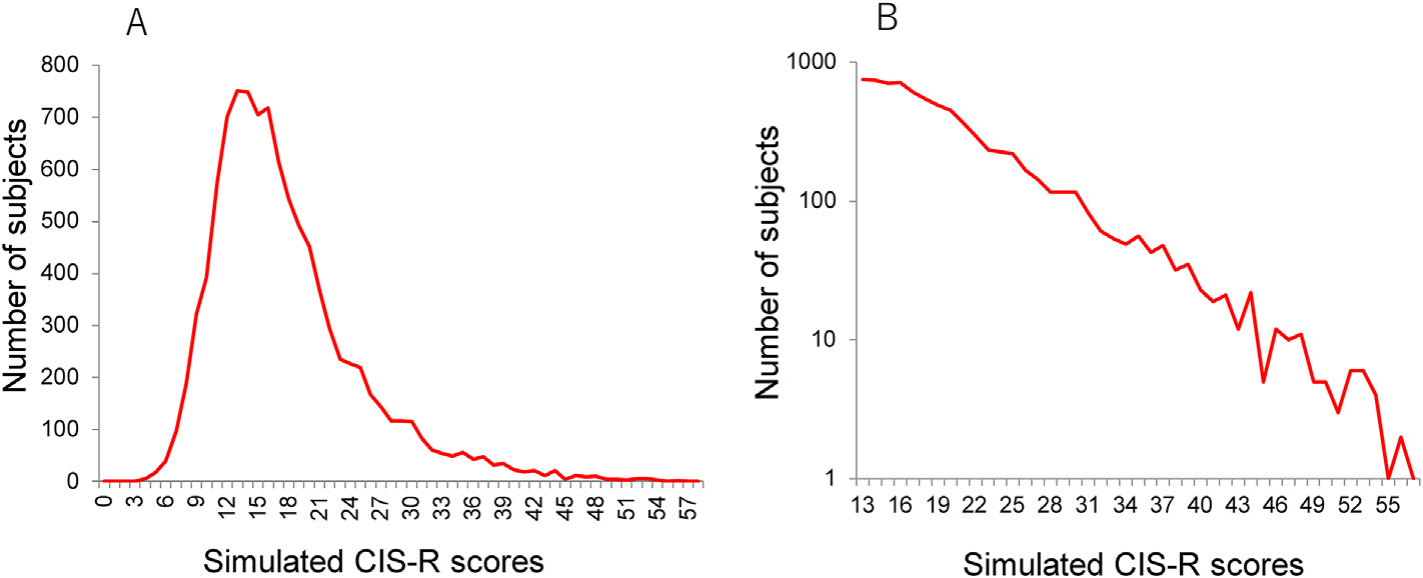
Distribution for simulated total scores on a normal scale (A) and a log-normal scale (B). Exponential distribution with a parameter of λ = 1 is set for the latent trait of depression, and a normal distribution with a standard deviation of 2 is used for the distribution of depressive symptom thresholds. Simulated total scores exhibit a right-skewed distribution (A). On a log-normal scale, the right tail of the distribution follows a linear pattern (B). Image credit: *BMC Research Notes*, https://doi.org/10.1186/s13104-017-2937-6.

As a result, when the severity of depression was set to an exponential distribution, and the thresholds for all 57 items were set to normal distributions with a certain degree of standard deviation, simulated CIS-R scores exhibited a unimodal and right-skewed distribution (Figure 7A) [23]. On a log-normal scale, the distributions for simulated CIS-R scores followed a linear pattern, except for the lower end of the distribution (Figure 7B). Furthermore, when the latent trait of depression was set to a normal distribution, the distributions for simulated CIS-R scores followed a normal distribution, except for the lower end of the distribution. These findings suggest that the model explains how the total score distribution reflects the distributional pattern of an individual's latent trait of depression.

Moreover, we investigated the relationship between an individual's latent trait of depression and simulated total scores [23]. Consequently, there was a linear relationship with a large effect size between them (*r*^2^ = 0.89). These findings suggest that the distribution of total scores on a depressive symptom scale reflects the distribution of an individual's latent trait of depression because of the strong linear relationship between an individual's latent trait and total scores.

Although a binary CIS-R questionnaire (0–1) was used for the simulation study, this process model is applicable to other scales with a greater number of response options. For example, in the case of the CES-D (0-1-2-3), each item is scored using four response options, indicating there are three thresholds for each item. Since the CES-D includes 16 depressive symptom items, it is expected to have 48 thresholds within a unidimensional latent trait. In other words, a four-point scale consisting of 16 items is equivalent to a binary scale consisting of 48 items from the viewpoint of a unidimensional latent trait. Since all depression rating scales are structured in the same way in terms of a single latent trait, total scores on a depression rating scale would follow an exponential distribution regardless of the number of response options used for each item.

### 3.4 Mechanisms for the specific pattern of item responses

As shown in Figures 3 and 5, responses to depressive symptom items exhibit a common mathematical pattern in a general population. The mechanisms that enable such a pattern of item response options can be speculated upon.

First, the specific pattern of item responses appears to be linked to the exponential pattern of the total score distribution because the specific pattern of item responses occurs only when the distribution of total scores approximates an exponential distribution [11, 12, 13]. Thus, it is necessary to elucidate how a probability of each response option is generated in terms of the total score distribution.

**Figure 8:**
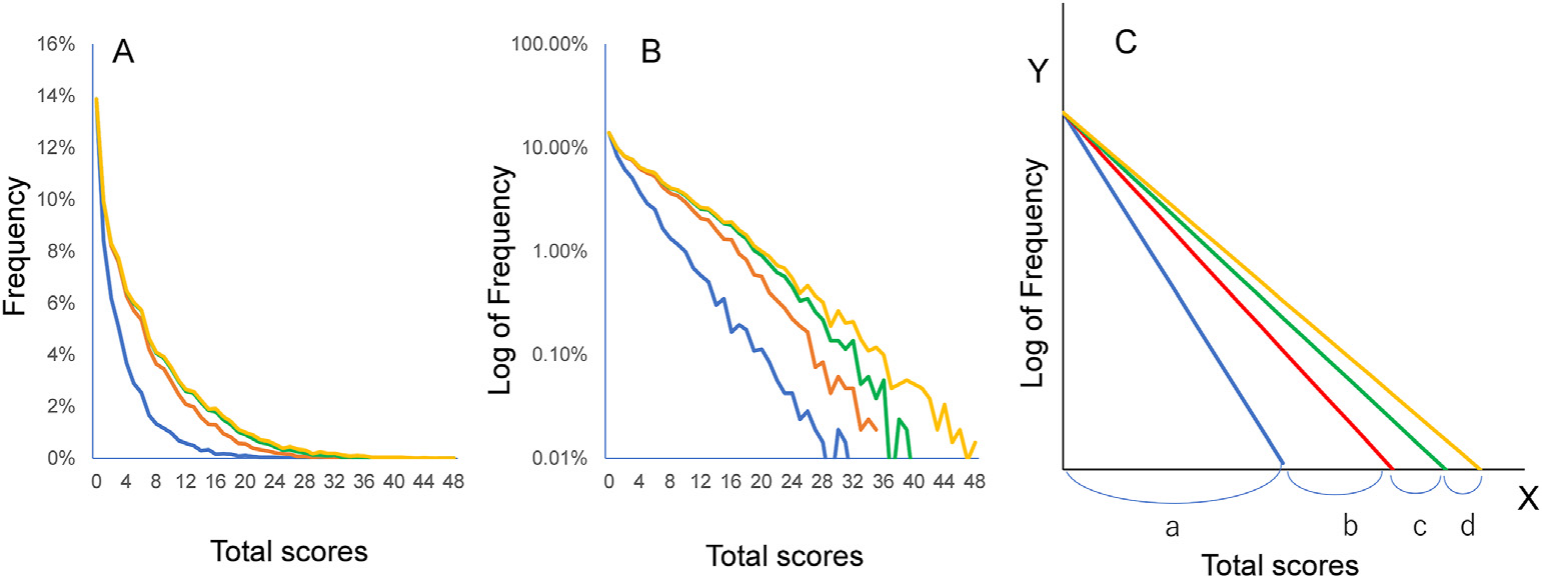
The distribution of total scores of 16 items (yellow lines) and the boundary curves of the next score for item 1 on a normal scale (A) and a log-normal scale (B). Blue, red, and green lines represent the boundary curves between scores 0 and 1, scores 1 and 2, and scores 2 and 3, respectively. The boundary curve of adjacent scores for item 1 exhibited a right-skewed distribution on a normal scale (A). On a log-normal scale, the boundary curves exhibit a linear pattern (B). Figure C is a simplified diagram for Figure B. Each area divided by the boundary curves forms a triangle on a log-normal scale. Each base of the four triangles is expressed as a, b, c, and d (C). Image credit: *PeerJ*, https://doi.org/10.7717/peerj.2566/fig-1.

In an analysis of CES-D data from a Japanese national survey, we investigated how the total score distribution was divided by the item scores of each depressive symptom [24]. Figure 8 depicts the distribution of total scores for 16 depressive symptom items (yellow line) on the CES-D and the boundary curves (blue line, red line, and green line) [24]. The blue, red, and green lines represent the boundaries among “rarely” (score = 0), “a little of the time” (score = 1), “occasionally” (score = 2), and “all of the time” (score = 3). The boundary curve of adjacent scores for item 1 (“bothered by things that usually don't”) exhibited a right-skewed distribution on a normal scale (Figure 8A). Four areas divided by the boundary curves correspond to each probability of “rarely,” “a little of the time,” “occasionally,” and “all of the time.” Notably, the boundary curves exhibit a linear pattern on a log-normal scale (Figure 8B). All boundary curves demonstrated such an exponential pattern for the remaining 15 depressive symptoms [24]. These findings agree with the process model of how the individual's latent trait of depression is processed into an item score (Figure 6). Theoretically, the boundary curve can be expressed as the product of the frequency of the latent trait of depression and the response rate of each response option. Our group's previous study mathematically proved that the boundary curves approximate an exponential pattern when the frequency of the latent trait of depression follows an exponential distribution and the response rate of each response option follows a cumulative distribution function of normal distribution [24]. The mathematical explanation has been reported in detail elsewhere [24].

Figure 8C is a simplified diagram of the boundary curves of adjacent option categories for a given symptom on a lognormal scale. Each area divided by the boundary curves forms a triangle on a log-normal scale. As the four triangles share the same “height,” the relationship of the four areas depends on each “base” of the four triangles (a, b, c, and d, in Figure 8C). Furthermore, as the four triangles share the same “height,” log-transformation of the Y-axis has little effect on the relationship of each “base” among the four response categories. Consequently, the relationship of each area among response categories (Figure 8A) corresponds to the relationship of each “base” of the triangles (a, b, c, and d, in Figure 8C). If the relationship of each distance for “b,” “c,” and “d” is proportional across all depressive items, the specific pattern of item responses will occur. Taken together, the findings suggest that the characteristic pattern of item responses occurs only when total scores approximate an exponential distribution. Conversely, the specific pattern of item responses will not occur when a latent trait of the measured object follows a normal distribution.

## 4 Discussion

This review has provided evidence that item scores and total scores on depression rating scales exhibit common distribution shapes in a general population and discussed how the distribution shapes are generated. To explain the mathematical pattern of item response and total score distributions, two conditions are assumed: (1) each individual's latent degree of depression forms an exponential distribution in a general population, and (2) the threshold of each depressive symptom forms a normal distribution.

Regarding total scores, previous simulation research has suggested that total scores on depression rating scales reflect a distributional pattern of latent traits of depression through a strong linear relationship between an individual's degree of a latent trait of depression and total scores [23]. Goldstein and Hersen stated that, to achieve an interval scale, equal differences on a scale must correspond to equal differences in the natural variable [36]. A strong linear relationship broadly satisfies this condition, suggesting that total scores on depressive symptom scales are not ordinal, but rather approximately interval in nature. Strictly speaking, since the linear relationship is not perfect, total scores on depressive symptom scales are imperfect interval data [37].

Of note, there is a long-running issue regarding whether Likert-type scales can be treated as interval scales (14–16). According to the scales of measurement theory (Stevens, 1946), Likert-type scale data are ordinal data because the interval between response categories is not equal (17). Researchers who espouse Steven's view claim that Likert-type scale data should not be treated as interval data, indicating that mathematical operations such as addition, subtraction, multiplication, and division are not applicable to them (18). On the other hand, a considerable number of researchers assert that, although item scores appear not to be interval, total scores on Likert-type scales are interval in nature (19). In fact, in the field of psychology and psychiatry, total scores on depressive symptom scales are widely treated as interval data (20). Previous simulation research supports the prevailing view that total scores on a Likert type scale tend toward interval data [23].

Regarding item scores, an analysis of boundary curves, which divide the distribution of total scores by each item score, revealed that there is a complicated relationship of each range among the response options on a depression rating scale. Moreover, the analysis of boundary curves suggests that the characteristic pattern of item responses occurs only when total scores approximate an exponential distribution. As shown in Figure 4, there is a trade-off relationship of each distance between the lower end option and the adjacent option, and a proportional relationship of each distance between all response options, except for the lower end option, across all items. Taken together, these findings suggest that item scores on a depression rating scale are not interval in nature.

The reason the relationship between response options differs according to the position of response options can be speculated upon. Practically, respondents follow a pattern when responding to depression rating questionnaires, such as the CES-D. First, they consider whether a specific symptom is present. If the symptom is absent, it is regarded as “rarely.” If the symptom is present, the degree of the symptom is quantified according to the remaining response options, such as “a little of the time,” “occasionally,” or “all of the time.” This two-step process could create a condition where the relationship between response options is different between the lower-end option and the remaining response options. Further research is necessary to clarify whether the relationship of each distance between the remaining options is proportional across all depressive symptom items.

There are some limitations to the process model presented in Figure 6. First, although a previous simulation study suggested that total scores on the depression scales reflect a distributional pattern of a latent trait of depression through a strong linear relationship between an individual's degree of a latent trait of depression and the total score, the findings should be interpreted with caution because of the parameters specified in the simulation [23]. Therefore, to generalize the findings, a mathematical proof for the findings is necessary. Next, although the assumption that a latent trait of depression follows an exponential distribution helps explain the mathematical pattern of item response and total score distributions, the reason that a latent trait of depression follows an exponential distribution remains unclear. Generally, an exponential distribution occurs when total stability and individual exchange are observed together, such as with financial income and maximum entropy [16, 38]. Further research is needed to clarify the mechanisms underlying the exponential pattern of a latent trait.

The major limitation of this review is the lack of extensive information on the distribution patterns of item responses ant total scores in various populations at different times. Extensive research needs to be undertaken to generalize the findings to different time periods, settings, and populations. In addition, although recent research has demonstrated that the total score distributions follow an exponential pattern except at the lower end of the distribution in the general population, we have not identified the mathematical pattern at this lower end. To develop a mathematical model for total score distributions on such scales, further research is needed to identify the mathematical pattern at the lower end of the distribution.

Despite these limitations, recent research shows the evidence that item scores and total scores on depression rating scales exhibit common distribution patterns in a general population. As noted in the Introduction, statistical procedures that assume a normal distribution are often used to analyze the depression scale data in epidemiological studies. According to the evidence collected so far, statistical procedures assuming normality may require careful consideration when analyzing such data. Furthermore, it is noteworthy that the exponential pattern covers a wide range of total scores beyond the cut-off points for clinical depression (Figure 1). These findings raise the possibility that depression is better conceptualized as a continuously distributed syndrome rather than as a discrete diagnostic entity [19]. Generally, the fact that intelligent test scores follow a normal distribution has provided a deep insight into the structure of intelligence [39]. In the same manner, the fact that item scores and total scores on depression rating scales exhibit common distribution patterns in the general population may shed new light on the mechanisms of depressive symptoms.

In conclusion, recent research has demonstrated that the distribution of item scores and total scores on depressive symptom scales exhibit a common mathematical shape in a general population. To explain the mathematical pattern of item response and total score distributions, the author assumed that a latent trait of depression follows an exponential distribution. This assumption helps explain the distributional patterns of item responses and total scores on a depressive symptom scale. Furthermore, these findings suggest that the mathematical pattern of item response and total score distributions on a depression rating scale may serve as evidence of the level of measurement.

## Declarations

### Author contribution statement

All authors listed have significantly contributed to the development and the writing of this article.

### Funding statement

This work was supported by 10.13039/501100001691Japan Society for the Promotion of Science (18K03145).

### Data availability statement

Data included in article.

### Declaration of interests statement

The authors declare no conflict of interest.

### Additional information

No additional information is available for this paper.
